# P-2067. Antibody Response in People Living with HIV on Effective ART: A Comparison of CD4 T-cell counts >200 versus ≤200 cells/mm³ Following a Bivalent mRNA COVID-19 Vaccine Booster, with Evaluation of the Correlation Between Anti-RBD IgG (Anti-Receptor Binding Domain Immunoglobulin) and sVNT (SARS-CoV-2 Surrogate Virus Neutralization Test)

**DOI:** 10.1093/ofid/ofae631.2223

**Published:** 2025-01-29

**Authors:** Napon Hiranburana, Opass Putcharoen, Nattakarn Thippamom, Stephen J Kerr, Supaporn Wacharapluesadee, Anchalee Avihingsanon

**Affiliations:** Division of Infectious Diseases, Department of Medicine, Faculty of Medicine, Chulalongkorn University, Bangkok, Thailand, Bangkok, Krung Thep, Thailand; Division of Infectious Disease, Department of Medicine, Faculty of Medicine, Chulalongkorn University, Krungthep, Krung Thep, Thailand; Thai Red Cross Emerging Infectious Disease Clinical Center, Chulalongkorn hospital, Bangkok, Krung Thep, Thailand; Chulalongkorn University, Pathumwan, Krung Thep, Thailand; Emerging infectious diseases clinical center, King Chulalongkorn Memorial Hospital, Pathumwan, Krung Thep, Thailand; HIV-NAT, Thai Red Cross AIDS Research Centre, Bangkok, Krung Thep, Thailand

## Abstract

**Background:**

**Despite protection against severe COVID-19 and SARS-CoV-2 variants in the post-pandemic era, the importance of mRNA booster doses may be underestimated. We assessed the immunogenicity of bivalent original/Omicron BA.4-5 COVID-19 vaccine booster in PLWH by immunosuppression level.**

**Table 1:**
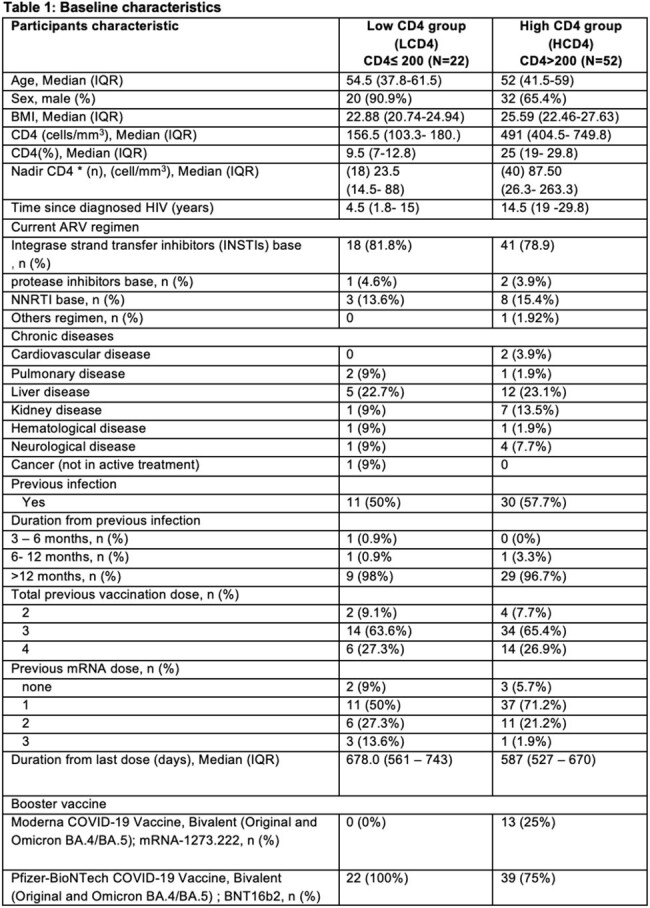
Baseline Characteristics of People Living With Human Immunodeficiency Virus (n = 74) at the time of administration of a bivalent original/omicron BA.4-5 mRNA vaccine booster dose divided into the low CD4 group (CD4 T-cell levels ≤200 cells/mm³) and high CD4 group (CD4 T-cell levels >200 cells/mm³). Abbreviations: HIV, human immunodeficiency virus; IQR, interquartile range; BMI, Body mass Index; ARV, antiretroviral; NNRTI, Non-Nucleoside Reverse Transcriptase Inhibitor

**Methods:**

This prospective cohort study enrolled participants aged ≥18 years, on stable ART, with ≥2 doses of COVID-19 vaccine, at King Chulalongkorn Memorial Hospital and HIV Netherlands Australia Thailand Research Collaboration. Exclusion criteria were virologic failure, previous immunosuppressant or monoclonal antibody use, recent vaccination, or SARS-CoV-2 infection. We evaluated anti-RBD IgG and sVNT against SARS-CoV-2 variants, before and 4 weeks after bivalent original/Omicron BA.4-5 mRNA vaccine booster. Primary outcomes were pre- and post-vaccination geometric mean titres (GMT) and post-vaccination GM ratios (GMR) of anti-RBD IgG in PLWH with CD4 T-cell levels ≤200 vs. >200 cells/mm³. Secondary outcome was the relationship between variant-specific sVNT and anti-RBD IgG.

**Figure 1:**
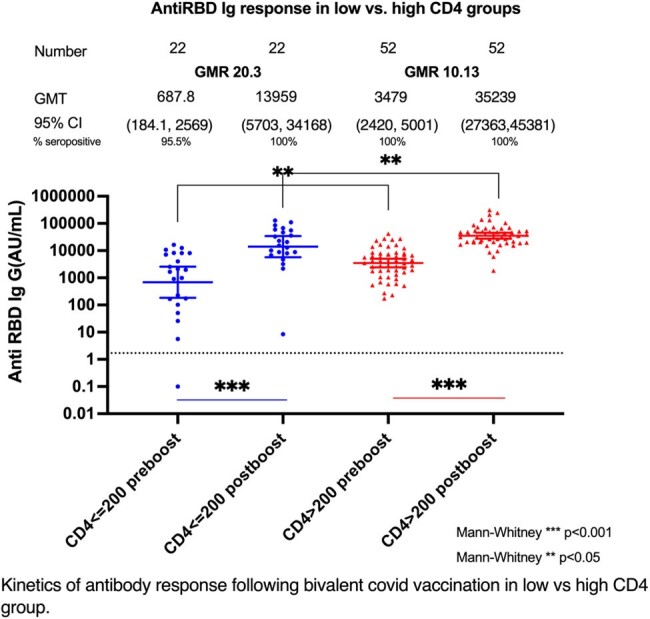
Kinetic of anti RBD Ig response after bivalent covid vaccine booster in patients with CD4 ≤ 200 cells/mm3 versus CD4 > 200 cells/mm³ Geometric mean titres (GMT) with 95% confidence interval (CI) of anti-RBD total Ig levels in CD4 ≤ 200 cells/mm3 and CD4 > 200 cells/mm³ group., Time points preboost: the day of vaccination; post boost, 4-week post vaccination; *** p<0.001; ** p<0.05 The cut off for seropositive from The SARS-CoV-2 IgG II Quant assay is log Anti RBD Ig 1.7 AU/mL. (50 AU/mL)

**Results:**

Of 74 participants, 22 (29.7%) had CD4 ≤200 and 52 (70.3%) had CD4 >200 cells/mm³. In the low CD4 group, median time since primary vaccination was 678 days; pre-and post-boost anti-RBD IgG GMT was 687 AU/mL and 13,959 AU/mL respectively (GMR increase = 20.3 (95%CI 7.3 to 56.2; P< 0.001). In the higher CD4 group, median time since primary vaccination was 587 days; pre- and post-boost GMTs were 3,479 and 35,238 AU/mL respectively (GMR increase =10.1 (95%CI 7.5 -13.7); P< 0.001) (Figure 1). Anti-RBD Ig titer correlated strongly with post-boost sVNT of Ancestral and BA.5 (vaccinated strain), XBB1.16, and XBB1.5 (circulating strains). Predicted sVNT inhibition was >40% for all strains at 4 log10 antiRBD IgG AU/mL (Figure 3).

**Figure 2:**
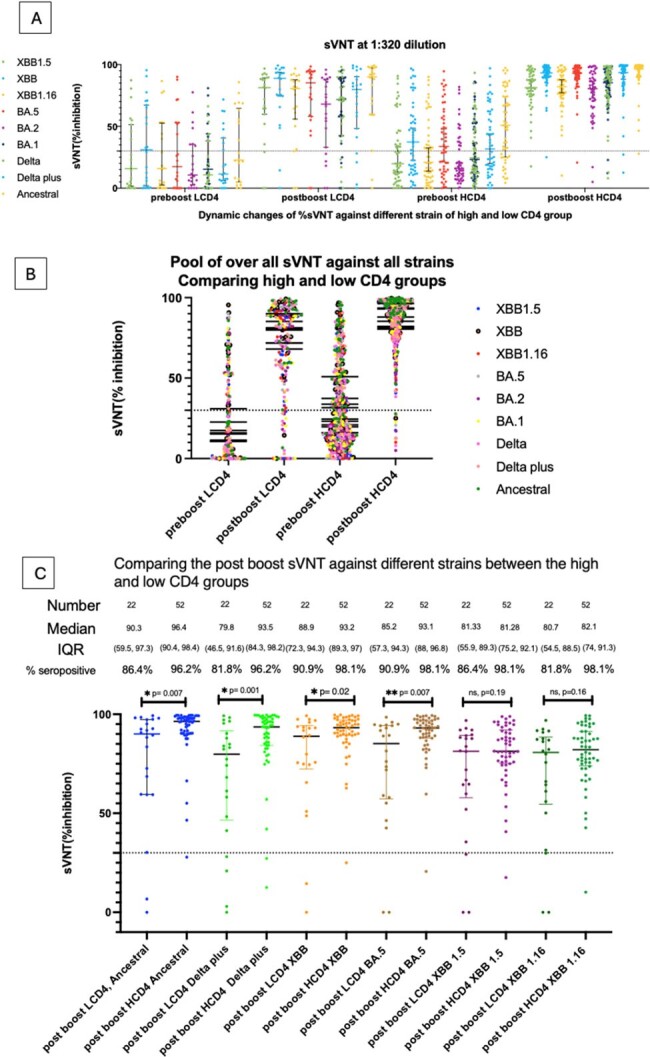
A-C: Dynamic changes of sVNT (% inhibition) with different SARS-CoV-2 variants in high and low CD4 group. Figure 2A: Dynamic change of sVNT (% inhibition) against different strains comparing high CD4 group (HCD4; CD4 ≤ 200 cells/mm³ ) and low CD4 group (LCD4; CD4 > 200 cells/mm³ ) Figure 2B: Pool kinetic response of sVNT (% inhibition) against concerning Omicron at the time of study, Delta, and Ancestral strains Figure 2C : sVNT (% inhibition) of post booster samples in different strains comparing high CD4 group (HCD4; CD4 ≤ 200 cells/mm³ ) and low CD4 group (LCD4; CD4 > 200 cells/mm³ ) Abbreviations: IQR, interquartile range. The cut off for seropositive from sVNT is 40% inhibition.

**Conclusion:**

Participants in our low and higher CD4 groups had significant increases in anti-RBD IgG, and neutralization antibodies with cross-reactivity against Omicron variants post-boost. This supports the importance of annual mRNA vaccine boosters for PLWH, especially those with low CD4 counts. Further research is needed to determine the optimal anti-RBD IgG level for adequate neutralization and assess the time course of immune protection to refine vaccination schedules in PLWH.

**Figure 3:**
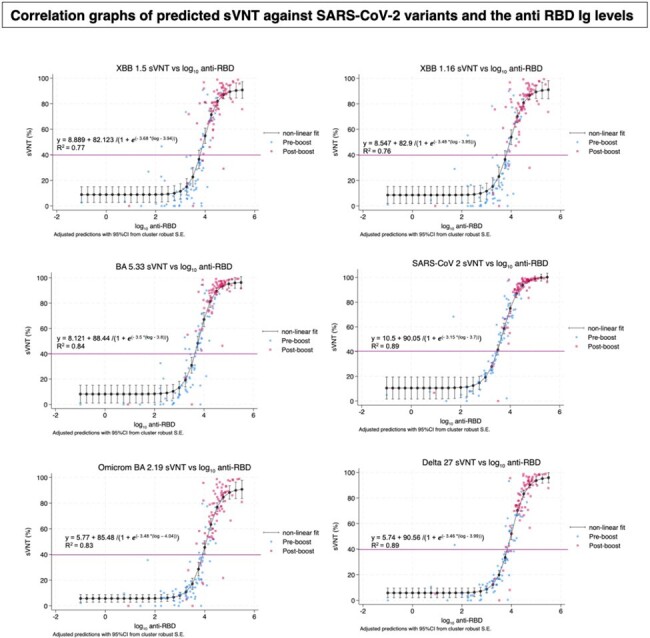
Correlation of predicted sVNT against XBB1.5, XBB1.16, BA.5, BA.2, Delta, Ancestral strain and log Anti RBD Ig titers using non-linear regression analysis.

Sera samples from individuals from pre-boost (blue) and at 4 weeks post-boost (red) with bivalent covid vaccine were tested with anti-RBD IgG and sVNT against XBB1.5, XBB1.16, BA.5, BA.2, Delta, and Ancestral strain.

Predicted sVNT level, using non-linear regression analysis with cluster-robust standard errors (N = 148) against different strains were based on the log anti RBD titer. The y axis represents percent inhibition of sVNT against different strains. The x axis represents log 10 scale of anti-RBD Ig titer (AU/mL).

The purple lines indicating sVNT cut off at 40%.

The r-square was calculated according to the non-linear equation using STATA v.17.0 software.

(The minimum log AntiRBD Ig required to achieve 40% sVNT was 3.75 for the ancestral/BA.5 strain (vaccinated strain) and 4 for the XBB1.5, XBB1.16, BA.2, and Delta strains.)

**Disclosures:**

All Authors: No reported disclosures

